# Supplementary material for: Causal Relationship between Aging and Anorexia Nervosa: A White-Matter-Microstructure-Mediated Mendelian Randomization Analysis
Source: Biomedicines. 2024 Aug 16;12(8):1874. doi: 10.3390/biomedicines12081874 (PMC11351342; doi:10.3390/biomedicines12081874)
Supplement: Supplementary file 1 [file biomedicines-12-01874-s001.zip › Supplementary Figure/Figure S8/Leaveoneout plot of MR analysis for TL to ubm-b-1960.pdf]

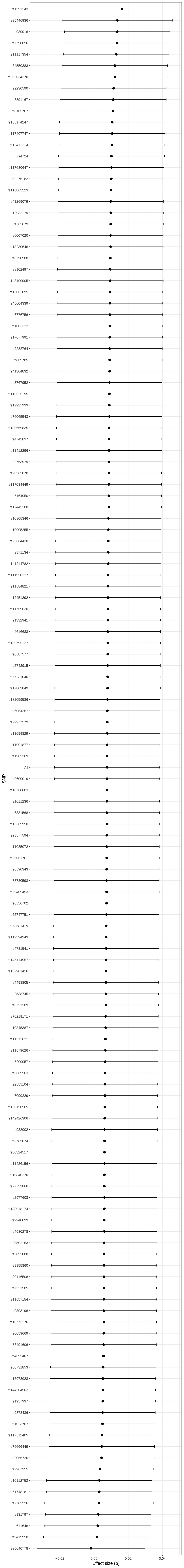
rs1291143
rs35446936
rs939916
rs7790856
rs11117354
rs34550383
rs202034370
rs2230590
rs3891167
rs8105767
rs185174247
rs117407747
rs12412214
rs4724
rs117630647
rs2276182
rs116863223
rs41269078
rs12932179
rs782679
rs6007020
rs13230646
rs6790988
rs8102497
rs143190905
rs13062095
rs45604339
rs6776756
rs1003322
rs17677991
rs2282764
rs869785
rs41304832
rs3767952
rs113525195
rs12925933
rs76065543
rs139669835
rs4743037
rs11412296
rs2763979
rs28363070
rs117034449
rs7164950
rs17445108
rs10805346
rs10905255
rs75664430
rs871134
rs141214782
rs111950327
rs11584821
rs12451892
rs11769630
rs1332941
rs4616688
rs139795227
rs6587577
rs5742915
rs77231040
rs17803849
rs182059586
rs6054257
rs79977579
rs11699829
rs11991877
rs1985369
All
rs9600019
rs10768683
rs1611236
rs6881568
rs12369950
rs28577594
rs11085072
rs56061761
rs6590343
rs73730598
rs59409453
rs6536702
rs55747751
rs73581419
rs112394943
rs4731541
rs145114957
rs137901416
rs4498805
rs2538745
rs6751209
rs76219171
rs10845387
rs11212631
rs11579626
rs7209057
rs6669563
rs2555104
rs7099229
rs150150565
rs142426306
rs932002
rs3785074
rs80324517
rs11426156
rs10840270
rs77732866
rs2977608
rs188918174
rs9940099
rs4530278
rs28502153
rs3093888
rs9955360
rs80116508
rs7221585
rs11557154
rs9398196
rs10773176
rs6659669
rs78491606
rs4695407
rs66731853
rs16978028
rs144204502
rs1957937
rs9878436
rs1023767
rs117512405
rs76666449
rs2056726
rs2987355
rs10112752
rs61748181
rs7705526
rs131797
rs611646
rs9419958
rs35640778
SNP
-0.02
0.00
0.02
0.04
Effect size (b)
